# Utilizing Apple Vision Pro for Mindfulness Intervention to Reduce Pain and Anxiety in a Hospitalized Patient With Mixed Connective Tissue Disease: A Case Report

**DOI:** 10.7759/cureus.107759

**Published:** 2026-04-26

**Authors:** Pouya Azar, James Wong, Mohammadali Nikoo, Victor W Li, Jessica Machado, Tam To, Christopher Haslam, Rodney Mullen, Borna Noureddin

**Affiliations:** 1 Psychiatry, Vancouver General Hospital, Vancouver, CAN; 2 Psychiatry, University of British Columbia, Faculty of Medicine, Vancouver, CAN; 3 Computing, British Columbia Institute of Technology, Burnaby, CAN

**Keywords:** anxiety, augmented reality, case report, opioid, pain, virtual reality

## Abstract

Developing and evaluating non-pharmacological treatments is critical for optimizing pain management and reducing dependence on opioids, as well as mitigating opioid-induced adverse effects. One promising approach involves the use of virtual reality (VR)- and augmented reality (AR)-guided mindfulness techniques. We examined the clinical utility of the Mindfulness app on the Apple Vision Pro VR/AR headset in managing pain and anxiety in a hospitalized patient with mixed connective tissue disease.

This case study presents a 22-year-old woman experiencing progressive acute-on-chronic pain and weakness, significantly impairing her mobility due to mixed connective tissue disease. The case report evaluated the effectiveness of a built-in Mindfulness app provided through AR on the Vision Pro. Conducted over 48 hours, the study assessed pain, anxiety, and as-needed medication usage before, during, and after the intervention for a single subject.

The patient participated in six intervention sessions, each averaging 59.67 minutes in duration. The Mindfulness app on the device reduced pain from an average of 4.67/10 pre-intervention to 1.83/10 post-intervention (p=0.03) and anxiety from an average of 6.5/10 pre-intervention to 2.83/10 post-intervention (p=0.03). The patient reported an overall positive experience with a motivation to utilize mindfulness interventions further in the future. The patient experienced some discomfort with the device due to its suboptimal fit and tolerable eye strain with prolonged use.

Utilizing Apple Vision Pro to deliver mindfulness interventions could serve as a viable non-pharmacological treatment modality for pain. Further research with adequately powered larger sample sizes and controlled designs is recommended to further study such interventions.

## Introduction

Chronic pain affects nearly eight million people in Canada, approximately one in five Canadians [1]. It is often persistent and complex and can significantly impair physical functioning, mental health, and quality of life. The condition has been commonly managed with pharmacological therapies, including opioids, raising concerns about overprescription and opioid-related harms. Contemporary clinical guidelines recommend the judicious prescription of opioids as modern data indicate that 4-6% of those misusing prescription opioids switch to heroin and 80% of those using heroin originally misused prescription opioids [2-4]. Canadian and American prescription opioid consumption rates remain among the highest in the world [5]. The development and evaluation of non-opioid treatments for pain management, including non-pharmacological treatments, are thus an important avenue.

Mindfulness is a practice characterized by bringing attention to the present moment through awareness and acceptance of thoughts, feelings, bodily sensations, and surroundings, serving as a non-pharmacological adjunct shown in numerous studies to be effective in managing pain, reducing anxiety, and increasing overall function [6-8], thereby potentially reducing opiate use in such settings. However, it is rarely applied in acute medical settings. Recent technological advancements have enabled immersive mindfulness solutions delivered through virtual reality (VR) and augmented reality (AR) modalities that can be used for people inexperienced in mindfulness without a human therapist. Previous research has studied the use of VR-/AR-guided mindfulness for the management of psychological and pain symptoms, with interactivity, multisensory delivery, and immersion identified as favourable features [9]. These approaches are argued to be more engaging and cost-efficient and have a strong potential for scalability compared to the traditional approach [10].

The Apple Vision Pro is a new VR/AR platform with complex sensor and audiovisual technologies to deliver an integrated immersive experience that may augment mindfulness practice and its effectiveness for the user. Collectively, these features enhance perceptual realism, contributing to a highly immersive user experience. There has been limited evidence on the feasibility and clinical impact of using mixed reality devices, such as the Apple Vision Pro, to deliver mindfulness interventions for pain for patients with complex autoimmune and connective tissue diseases [11,12]. These patients experience persistent pain that is difficult to manage pharmacologically, and opioids carry risks. Driven by the aim to fill this gap and improve pain management with novel non-pharmacological approaches, we used the Mindfulness app on the Vision Pro to examine its utility in a hospitalized patient with severe acute-on-chronic pain.

## Case presentation

Patient information

This report describes a 22-year-old woman in her final term of nursing school. She had a medical history of a connective tissue disorder, likely polymyositis, diagnosed two years prior, and a history of shingles at the L2L3 dermatome one year prior. She also reported a history of Raynaud's phenomenon before her polymyositis diagnosis two years prior. She reported a history of fatigue and bilateral burning leg pain, primarily localized to the quadriceps with occasional involvement of the calves. She previously underwent a trial of chloroquine, but was not on this medication at the time of presentation. She did not recall any previous trials of pain medications aside from sporadic use of acetaminophen for her leg pain. Otherwise, she was healthy, was not taking any medications at home, and denied any substance use, including alcohol and nicotine. She also denied any family history of chronic pain, substance use disorder, or mental illness.

She had three emergency department visits over a span of 16 days, eventually leading to her admission due to progressive acute-on-chronic pain and weakness, significantly affecting her mobility. In the background of her chronic leg pain, she reported experiencing a shooting, stinging sensation running down her legs, accompanied by reduced sensation starting from her feet and ascending into her abdomen. She also reported paresthesia extending into her lower abdomen and chest, along with pain in her lower face. During admission, neurology evaluated her with a diagnostic impression of asymmetric polyneuropathy involving both sensory and motor components, with elements of mononeuritis multiplex. She was started on steroids and intravenous immunoglobulin (IVIG) with some improvement. She was then transferred to our hospital for further workup and diagnostic clarification.

She was consulted by our hospital's inpatient pain consult team. Upon transfer, she was on a combination of pain medications including ketorolac 10 mg IV/IM three times a day (TID), gabapentin 300 mg orally (PO) TID, nortriptyline 20 mg PO at bedtime, ketamine 50 mg PO every six hours, acetaminophen 320 mg PO every six hours (combined with ketamine), and a recent start of methadone 1 mg PO TID. She also had as-needed (PRN) medications available, including gabapentin, ketorolac, hydromorphone, acetaminophen, and ketamine. The initial plan was to optimize the dose of methadone, gradually taper off high-dose hydromorphone, initiate oral nonsteroidal anti-inflammatory drugs (NSAIDs) for pain management, reduce the dose of ketamine and Tylenol, and further optimize the dose of gabapentin based on her response and tolerability. Nortriptyline was discontinued to simplify her neuropathic medication regimen.

Clinical findings and course of admission

In terms of medications for her mixed connective tissue disease, she was on prednisone, rituximab, and IVIG. She was placed on ambrisentan and tadalafil for her pulmonary hypertension.

The patient's admissions were extended over several weeks. Notably, the patient experienced a five-day intensive care unit (ICU) admission early in her hospital stay, presenting with fever and hypotension, though pan cultures were negative. During her ICU stay, she had a significant decompensation episode, resulting in flash pulmonary edema and Takotsubo cardiomyopathy, necessitating a code blue. Consequently, her oral medications were held, and methadone was restarted at a lower dose.

After transferring out of the ICU, the patient's daily total hydromorphone usage remained unchanged despite repeated increases in methadone and the absence of any evidence of disease progression and ongoing treatment of the underlying disease. It was observed that she developed significant anxiety related to her health, given the rapid onset of her medical issues, ICU admission, and functional decline. This anxiety was hypothesized to be reinforcing her hydromorphone use. When discussed with the patient and her family, they agreed with this hypothesis. Although mindful interventions were suggested to address her anxiety, the patient did not engage with these interventions despite expressing interest.

Table 1 provides a summary of significant findings from physical examination and investigations during the admission.

**Table 1:** Summary of significant findings from physical examination and investigations during the admission EMG: electromyography; MRI: magnetic resonance imaging

Relevant examinations and investigations	Findings
Neurological exam	Normal cranial nerves except the following: neck flexors: 4/5 strength; light touch, pinprick, and temperature: reduced distally in the upper and lower extremities, especially over the dorsum of the feet, and patchy up to the lower thighs in the lower extremities and above the elbows in the upper extremities; vibration: normal at fingertips, absent at toes and ankles, and reduced at tibial plateaus (normal tone and muscle bulk); shoulder abduction: 4+/4-; elbow flexion: 4+/4; elbow extension: 4+/4-; wrist extension: 4+/1; finger flexors: 4+/4; extensor digitorum communis: 4+/1; finger abduction: 4+/1; abductor pollicis brevis: 4/1; thumb extensor: 4-/1; flexor pollicis longus: 4/1; hip flexion: 4-/4-; knee flexion: 5/5; knee extension: 5/5; ankle dorsiflexion: 2/4-; ankle plantarflexion: 4/4; extensor hallucis longus: 1/1; biceps: 2+; triceps: absent; brachioradialis: absent; patellae: absent; Achilles: absent; and plantar reflexes: downgoing
Electrodiagnostic studies	There is evidence of multiple asymmetric axonal peripheral neuropathies (i.e., mononeuritis multiplex). There were no features of demyelination. EMG revealed several areas of neurogenic changes but no definite signs of myopathy
MRI findings	Muscle edema involving the lower extremity adductor compartments bilaterally, the left vastus lateralis, and, to a lesser extent, the right long head biceps femoris. Findings are most in keeping with myositis. Normal MRI spine, brain, and brachial plexus
Muscle/nerve biopsy	The inflammatory myopathy does not have the pattern of a recognized subtype such as polymyositis, inclusion body myositis, immune-mediated necrotizing myositis, inflammatory myopathy with perimysial pathology, or dermatomyositis. The changes can be seen in systemic connective tissue disorders. There is invasion of the wall of epineurial arterioles by inflammatory cells, some of which resemble neutrophils. This is pathognomonic for small vessel vasculitis. This is accompanied by a severe axonopathy with the changes of acute Wallerian degeneration
Mitogen panel	ANA 1:1280, nucleolar profile positive with anti-Ro52 highly positive and scleroderma disease profile positive

Therapeutic interventions

The proposed intervention was the use of the built-in Mindfulness app provided through AR on the Vision Pro, with a planned duration of 48 hours, assessing pre-during-post scores on pain, anxiety, and PRN usage as outcomes. The Mindfulness app displays an expanding and contracting circle of flower petals to guide the user to focus on their breathing as a trainer guides them on a meditation. Written informed consent was obtained after discussing the intervention and its risks and potential benefits with the patient and her family. She was advised to record the duration of each session during the 48-hour window and to log pre- and post-session pain and anxiety levels using the Visual Analogue Scale (VAS) from 1 to 10. VAS has been previously used and validated for the assessment of anxiety [13,14] and pain [15,16]. We also conducted a post-intervention survey to assess the outcomes of the intervention (see Appendices). Data analysis was descriptive. IBM SPSS Statistics for Mac, Version 29.0.1.0 (IBM Corp., Armonk, New York, United States), was used to conduct the statistical tests.

**Figure 1:**
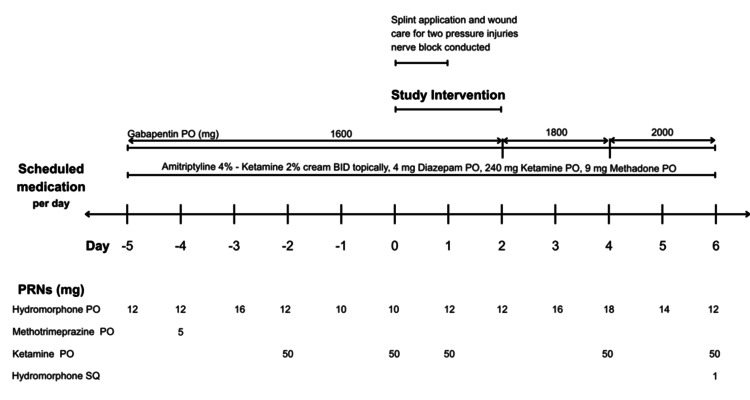
Summary of scheduled and PRN medication usage around the time of the intervention's administration PRN: as-needed

Prior to implementing the Vision Pro intervention, the patient and the team agreed not to make any further changes to her pain medication orders six days prior to the intervention to establish a baseline. We prescribed the device to be used approximately every three hours when awake, mimicking the pattern of her PRN hydromorphone. This approach was consistent with the family's understanding of the need for pain management intervention. We recommended that she use the device for at least 10 minutes, with the possibility of extending usage if tolerated. The patient participated in six sessions of the offered intervention over a 48-hour study period, with each session lasting an average of 59.67 minutes (range=28-80; standard deviation (SD)=22.6). Figure 1 provides a summary of scheduled and PRN medication usage around the time of the intervention's administration.

Follow-up, outcomes, and patient perspective

**Figure 2:**
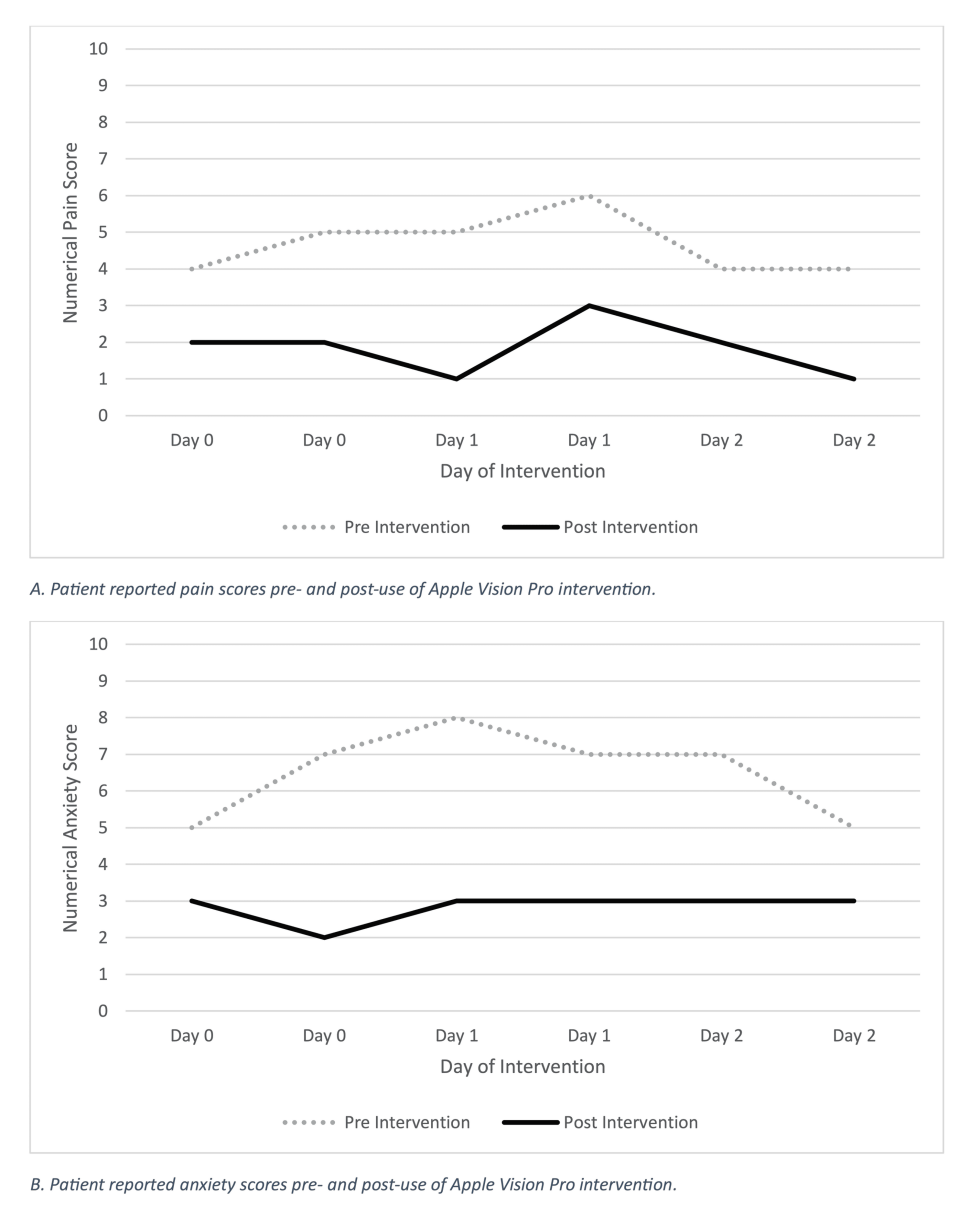
Trend of pre- and post-pain and anxiety VAS during the intervention The figure depicts the trend of pre- and post-pain and anxiety VAS during the intervention. VAS ranges from 0 to 10, where the higher scores indicate the greater the severity. For pain, 0 means no pain, and 10 means the worst pain possible. For anxiety, 0 means not anxious at all, and 10 indicates extremely anxious. VAS: Visual Analogue Scale

Figure 2 shows the pre- and post-pain and anxiety VAS. The patient's average pre-intervention pain was 4.67 out of 10 (range=4-6; SD=0.75), which decreased to 1.83 (range=1-3; SD=0.69) post-intervention. Her average anxiety score before the intervention was 6.5 (range=5-8; SD=1.12), which decreased to 2.83 (range=2-3; SD=0.35) after the intervention. The average daily usage of PRN hydromorphone was 12 mg in the week leading up to the intervention and remained 12 mg per day in the 48 hours leading up to the intervention. This compares to an average daily use of 11 mg during the 48-hour intervention period.

The patient reported that she would have preferred to use the Vision Pro more frequently, but she was busy with visits from multiple care providers. Regarding sleep during this period, she reported an improvement from her baseline as she was able to sleep without waking up for one night. The use of the Mindfulness app on the Apple Vision Pro had encouraged her to use other mindfulness applications in the future for managing her pain and anxiety, given the positive experience that she had with this device.

In the post-intervention questionnaire (see Appendices), she rated her overall satisfaction with the experience as 5 out of 5. She cited the following positive aspects of the experience. She enjoyed the calming and relaxing effect provided by the visuals of the floating flower petals that surrounded her, the guiding voice, and the background music. She felt like she was transported to a "different world". She also valued the device's capability to detect and time her breathing patterns, enhancing her mindfulness practice. Additionally, she appreciated the device's ability to "darken" the screen when concentration was necessary, helping to minimize distractions from external stimuli. Moreover, she found it beneficial when the guiding voice encouraged her to reflect on her day and let go of any negative thoughts, further aiding her relaxation and mental clarity. She rated 5/5 her desire to use the device again.

She cited the following as features which could be improved about the experience. She expressed a desire for more variety in the visuals, specifically mentioning that the only available option of flower petals was limiting. Additionally, she found that the guiding voice paused for too long at times, causing her mind to wander. The patient experienced discomfort with the device (3 out of 5) due to its fit being too large for her head and needing to wedge her glasses into the goggles for her myopia. She also reported eyestrain, rated 4/5 in severity (5 being no strain at all), which she nonetheless found manageable as it only emerged when using the device constantly for more than an hour.

## Discussion

This case report describes one of the earliest clinical uses of mindfulness interventions delivered through the Apple Vision Pro for acute pain management. This intervention reduced the levels of pain and anxiety post-intervention. The level of PRN hydromorphone usage did not change after the intervention. She had an overall positive experience with the device and was motivated to use it again; however, she reported some discomfort due to a suboptimal fit and manageable eyestrain.

Current understanding about the effectiveness of AR/VR technologies in the management of pain is limited by heterogeneous applications and durations of VR/AR treatment; despite this, there is growing evidence that VR/AR applications are effective in acute as well as chronic pain settings [17]. It is notable that in 97.8% of the included studies for acute pain, distraction was reported as the responsible mechanism [17]. In the present case, distraction ("transported to a different world") was a major component of the patient's experience alongside her reduction in pain scores. There is earlier evidence that distraction from painful stimuli is effective in reducing acute pain, regardless of the level of anxiety [18].

The effect of VR on pain-associated anxiety in the hospital setting was explored in a recent systematic review, which found that VR across many applications had mixed results for anxiolytic effects [19]. In contrast, the patient in this case had a substantial reduction in anxiety scores while using a guided mindfulness exercise, which is known to be effective for reducing anxiety [20]. It is difficult to determine whether the reductions in pain and anxiety are interrelated or if this is specific to mindfulness-based treatment. While mindfulness exercises delivered by VR have been found to be directly effective in chronic pain reduction [12], mindfulness-based interventions have lacked evidence for reducing pain severity or pain distress in acute pain [21], suggesting that mindfulness interventions likely need longer durations to have an effect on pain perception and impact. We can speculate that the high-resolution displays and the unique user experience of the Apple Vision Pro, compared with other headsets, may contribute to her positive experience, but further studies are needed to determine if these features result in any clinically significant difference compared to simpler and less expensive VR/AR platforms [22].

The patient did experience discomfort with the device due to its fit, and this likely would have negatively impacted the platform's effectiveness in reducing anxiety and discomfort. We were limited by possessing only one band, light seal, and light cushion which were not tailored for the patient, which contributed to the suboptimal fit. Furthermore, she required eyeglasses underneath the device as optical inserts were unavailable outside the United States at the time of the study. Despite the suboptimal fit, the patient experienced a high degree of satisfaction and encountered no issues with the headset's hand and eye tracking.

One limitation of this study was the inability to distinguish between the contributions of mindfulness and AR/VR on the observed improvement, which could be the subject of future research. However, previous studies have shown that engagement with self-help mindfulness interventions and a high attrition rate are barriers to their use [23]. In this case, it was observed that she did not engage with mindfulness interventions despite an expressed interest. There is preliminary evidence that using AR/VR can enhance engagement in digital applications [24]; however, more research is required to explore this hypothesis for the utilization of mindfulness interventions. Moreover, we were not able to rule out the placebo effect of our intervention, which could be further explored in future controlled studies. Future research should also examine whether the effects of the intervention on pain, anxiety, and PRN use persist after its application and determine the optimal frequency and period of the intervention. Additionally, future studies should utilize randomized controlled study designs and larger sample sizes to rigorously examine the effectiveness of the device, for instance, by comparing it with other VR/AR headsets and two-dimensional video-based interventions. The design of more personalized VR/AR mindfulness applications is also paramount, where users can curate the visuals and audio of their experiences. Biofeedback could provide further immersion, adapting to the user's bodily functions in real time, while multi-user mindfulness sessions could foster social cohesion.

## Conclusions

This case demonstrated that mindfulness interventions via Apple Vision Pro provided acute symptom relief during sessions and warrant further investigation to determine if effects persist and translate to reduced medication use. Although these results are encouraging, it is important to examine this device in more rigorous and larger studies. Investigating the outcomes of this intervention among several individuals in controlled settings, potentially administered over a longer period and using more feature-rich mindfulness interventions on this device or similar platforms, will help better understand its effectiveness and potential for broader clinical application.

## Appendices

**Table 2:** Post-intervention survey

How satisfied were you with the experience?				
1	2	3	4	5
Not at all				Very much
Was the headset comfortable?				
1	2	3	4	5
Not at all				Very much
Did the headset cause any strain on your eyes?				
1	2	3	4	5
Not at all				Very much
Would you want to use the headset again?				
1	2	3	4	5
Not at all				Very much
